# Examining How Ambidextrous Leadership Relates to Affective Commitment and Workplace Deviance Behavior of Employees: The Moderating Role of Supervisor–Subordinate Exchange *Guanxi*

**DOI:** 10.3390/ijerph17155500

**Published:** 2020-07-30

**Authors:** Mengying Wu, Rongsong Wang, Peixu He, Christophe Estay, Zubair Akram

**Affiliations:** 1 Glorious Sun School of Business and Management, Donghua University, Shanghai 20001, China; 17864192025@163.com; 2 Oriental Enterprise Management Research Center & Research Center of Business Management, Business School, Huaqiao University, Quanzhou 362021, China; hepeixu@hqu.edu.cn; 3 Strategy and Entrepreneurship Department, EM Normandie Business School, 30, rue de Richelieu, 76087 Le Havre CEDEX, France; estay.christophe@gmail.com; 4 School of Management and Economics, Beijing Institute of Technology, Beijing 100081, China; zubairakram91@yahoo.com

**Keywords:** ambidextrous leadership, affective commitment, supervisor-subordinate exchange *guanxi*, workplace deviance behavior

## Abstract

How to regulate employee conduct and engage them in high performance works actively and continuously has always been the important topic for organizations. Based on affective events theory and social exchange theory, a moderated mediating model was constructed with the affective commitment as mediator and the supervisor–subordinate exchange *guanxi* as moderator. Regression analyses and conditional indirect effects were tested by SPSS and PROCESS with 374 matched supervisor–subordinate pairs. The paper explores the moderated mechanism of supervisor-subordinate guanxi to the chain of “ambidextrous leadership–employee’s affective commitment–workplace deviance behavior.” The results showed that the affective commitment mediated the effect between ambidextrous leadership and employees’ workplace deviance behavior, and supervisor–subordinate exchange *guanxi* moderated the relationship between ambidextrous leadership and employees’ workplace deviance behavior but also moderated the mediating effect of affective commitment. The results have significances to improve human resource management practices and reduce the workplace deviance behavior of employees.

## 1. Introduction

Today, the acceleration of economic globalization, information networking, and resource intelligence leads to the intensification of global market competition [1], which forces organizations to focus on industrial upgrading, technological innovation, and management transform [2]. This causes workplace deviance to increase with the complexity of the business environment and social background. Among the many studies on organizational behavior, workplace deviance attracts more and more scholars’ and practitioners’ attention [2,3]. As a deliberate behavior of misconduct by employees, the potential harm of workplace deviance behavior is greater than its possible benefit for the organization [3]. For instance, a survey of 800 managers from 17 industries showed that workplace deviance behavior reduced employees’ hard work by 48%, quality of work by 38%, organizational commitment by 78%, and organizational performance by 66% [4]. The deviance behavior that goes beyond the bounds allowed by organizational norms could raise many potential problems, such as legal proceedings, employee turnover, inefficient production, and negative corporate public image [5]. These problems have increasingly become a predicament for managers to get rid of [6].

Prior research has found the positive outcomes of citizenship behaviors (i.e., positive voluntary behavior), but the exploration of negative voluntary behavior is still insufficient [5,7]. Placing emphasis on the positive does not mean that negative work behavior will be “erased.” To date, management support, organizational trust, ethical leadership, and other factors are found to motivate employees to engage in workplace citizenship behavior [8,9]. However, few studies have been conducted to control and predict those factors that contribute to workplace deviance behavior (negative voluntary behavior), especially concerning research on their driving motivation and management strategies from the interactive balance perspective of leadership style [10]. Although both deviance behavior and citizenship behavior are voluntary performances in the workplace, the mechanisms implied in their definitions and results are very different. Specifically, workplace deviance behavior refers to “voluntary behavior that violates significant organizational norms and in so doing threatens the wellbeing of an organization, its members, or both” [11,12], and workplace citizenship behavior is a voluntary extra-role behavior for promoting business growth and improving performance [13]. Therefore, extending prior studies on comparing the two variables, we further explore whether the conclusions of those studies on workplace citizenship behavior are applicable to workplace deviance behavior.

The extant research on employee workplace deviance behavior is not as extensive as that on organizational citizenship behavior. Our study aims to provide answers to the following two research questions: (1) what is the driving mechanism and the forming process of the employee workplace deviance behavior? and (2) How to intervene and control workplace deviance behavior and formulate reasonable management strategies? We analyzed prior literature and found that the main research model was based on social exchange theory [14], which emphasized that organizational injustice could damage the social exchange relationship between employees and the organizations, and employees undertook workplace deviance behavior to restore fairness and reduce losses [15]. Moreover, recent developments in the literature focus on the affective event interpretation model of employee workplace deviance [6,16]. The model suggests that employees’ behavior and performance are largely influenced by their emotions, which change every moment in their work. Different affective events (e.g., organizational justice or leadership) in the work context may lead employees to develop specific moods and emotions, which then bring out some sentiments and behaviors [17]. Affirmative emotions (such as happiness or confidence) are related to positive behavior such as organizational citizenship behavior, while passive sentiments (such as nervousness or depression) are related to negative behavior such as employee workplace deviance behavior [18]. The affective event interpretation model of employee workplace deviance is based on affective events theory (AET), which explains that employee emotions and moods may affect employee behavior directly or indirectly by influencing their attitudes [19]. Therefore, we aim to combine affective events theory with the latest research in the area to construct a chain of “event–affective–attitude–behavior” for revealing systematically the affective operation mechanisms of workplace deviance behavior.

## 2. Theory and Hypotheses

### 2.1. Theoretical Discussion

“Ambidexterity” is a concept that contains both opposition and unification. Duncan et al. [20] first introduced the concept into the field of management science to describe the dual ability of an organization in operating a business effectively and accommodating future changes. Some years later, March et al. [21] further introduced “ambidextrous” into the field of innovation management and divided ambidextrous innovation into exploratory innovation and exploitative innovation. As the motive force of organizational activities, the dilemma of how exploratory innovation coexists with exploitative innovation brought a climax to the research on ambidextrous ability. With the deepening of relevant research, ambidexterity intersects with technological innovation, organizational learning, strategic management, etc., and its connotation expands to the leadership field [22,23,24]. Bass [25] points out that transformational leadership inspires the creativity of employees through idealized charisma and identifies opportunities in the external environment. Transactional leadership ensures the order of organizational activities by setting clear rules and norms [26]. The “superposition effect” between transformational leadership and transactional leadership can balance and coordinate the relationship between two or more contradictory parties effectively and make adaptability adjustments according to the organizational changes [27]. The ambidextrous leadership formed by the combination of transformational leadership and transactional leadership breaks through the pressure of convention bondage and alleviates the confusion of cognitive diversity [28].

How to identify and determine leadership effectiveness is always the concerns of practitioners and scholars who are interested in leadership [15,29]. Effective leadership is conducive to understanding the process of interaction between leaders and employees [30]. However, with the increasing complexity, diversity, and competitiveness of the environment, it becomes a challenge to the leaders who manage the development of business [31]. Some scholars believe that ambidextrous leadership is composed of transformational leadership focusing on motivation and empowerment and transactional leadership focusing on task efficiency. Ambidextrous leadership can use flexible strategies according to changes to the external environment, management objects, and different purposes in order to meet the complex performance objectives and dynamic situational needs of organizations [32,33]. Ambidextrous leadership combines complementarity and convertibility, which has cognitive flexibility and behavioral complexity. In addition, ambidextrous leadership has the ability to manage conflicts by understanding employees’ perceptions and expectations while coping with contradictions [32]. Compared to the consistency of unidimensional leadership, the fundamental underpinning of ambidextrous leadership is that it focuses on the contingence of leadership according to the specific circumstance [34]. As a result, leadership effectiveness and organizational performance is achieved through increasing communication and exchange between leaders and employees, and the reduction of workplace deviance behavior [28,35].

What the literature suggests is that workplace stressors are a potential predictor of employees’ negative organizational behavior [36,37]. However, the influence of ambidextrous leadership on workplace deviance behavior and the mechanism implied in the affective cognitive experience on employee behavior has been underdeveloped in the literature. Our study thus explores the concept that affective commitment, which is defined as a psychological bond between individuals and organizations, may be an important mediator variable for “ambidextrous leadership–workplace deviance” [35,38]. Meanwhile, “*guanxi*” refers to having personal trust and a strong relationship with someone and can involve moral obligations and exchanging favors [39]. Considering that the cultural characteristics of Chinese society are guided by *guanxi* and the relationships with leaders are paid more attention by Chinese employees, the supervisor-subordinate exchange *guanxi* may have impact on the attitude, emotion and behavior orientation of employees [40]. According to affective events theory and the perspective of *guanxi* variable in Chinese culture [19,39], we integrate supervisor–subordinate exchange *guanxi* and the antecedents of workplace deviance behavior into the study of leadership style and employee negative behavior. Our research posits that supervisor–subordinate exchange *guanxi* may play an important moderating role in the path of “ambidextrous leadership–affective commitment–workplace deviance” [41]. The mediating effect of affective commitment on ambidextrous leadership and employee’s workplace deviance behavior is more significant in high-quality supervisor–subordinate exchange *guanxi*. In this paper, the affective driving model and managerial implications for employee workplace deviance behavior will be proposed through the theoretical view of affective commitment and supervisor–subordinate exchange *guanxi*. This study aims to seek the theoretical and practical methods to solve the widespread organizational problem of “inefficiencies in controlling and preventing employee workplace deviance behavior” and to construct a new normal mechanism for human resources management.

### 2.2. Ambidextrous Leadership and Workplace Deviance

There has long been interest in how leadership can affect employees [42,43]. Ambidextrous leadership is a special leadership with two different styles (transformational leadership and transactional leadership), and the ambidextrous aspects have a dialectical relationship with each other in the same direction [26]. In general, positive leadership can provide higher-quality products and more effective services to the organization. Both transformational leadership and transactional leadership cab improve employees’ performance and satisfaction, and reduce employee workplace deviance behavior [27,28]. Specifically, supervisors mainly take the following steps to prevent the negative behavior of employees in the workplace: (1) creating an organizational moral atmosphere, because the type and intensity of organizational moral atmosphere will affect employee workplace deviance behavior; (2) providing objectives and methods for employees, establishing trust relations within the organization through reasonable guidance and management by objectives, and establishing a constructive role in the organizational culture; and (3) aligning rewards and punishments to real performance [44,45]. Leadership plays an important role in the implementation of reward and punishment, and workplace deviance behavior is closely affected by a reward and punishment system [46]. Furthermore, the extant literature suggests that there is a positive correlation between transformational leadership and leadership effectiveness [47], while transactional leadership pays more attention to criticism, correction, and punishment of workplace deviance behavior. They can both suppress the negative impact of organization behavior effectively [26].

Ambidextrous leadership considers organizational management and employee development with paradoxical thinking, which has a significant impact on workplace deviance behavior [33]. Specifically, on the one hand, the literature suggests that transformational leadership is closely related to the positive motivation and employee behaviors in many cases, and its essence is to exchange the approaches and achieve any desired effects [48]. Transformational leadership stimulates employees to support organizational change through delegating power, establishing vision, and exalting ideals, and it encourages employees to take a different perspective and a reasonable way to address deviance [36]. It also trains employees to pursue noble sentiment through internal incentives to reduce the motivation of employee workplace deviance behavior [10]. On the other hand, transactional leadership uses strict rules and regulations, emphasizes work quantity and quality, promotes task orientation and economic performance, and mainly focuses on the relationship quality between supervisors and subordinates [49]. Walumbwa et al. [50] believe that transactional leadership has a positive impact on organizational citizenship behavior. When supervisors and subordinates trade on the principle of mutual approval, they are more likely to follow an implicit contractual relationship. It also reflects the negative effect of transactional leadership on workplace deviance behavior from another perspective. That is to say, employees are more likely to exhibit citizenship behavior under their approved contractual relationship, and form workplace deviance behavior under their unapproved contractual relationship [51]. Therefore, transactional leadership concentrates on task-oriented management, which has a great impact on the specific behavior of employees and can supervise and restrain their workplace deviance behaviors. As such, we propose that

**Hypothesis** **1.**  *Ambidextrous leadership has a negative impact on the workplace deviance behavior of employees.*

### 2.3. The Mediating Role of Affective Commitment

Affective commitment is the core dimension of organizational commitment [52]. It is an important variable that can satisfactorily forecast the effect on employee performance in the field of organizational behavior [53]. According to Cheng and Stockdale [54], Chinese culture differentiates itself from other cultures in that Chinese people put a high importance on “affection,” and it is crucial to maintain the relationship between individuals and organizations. In the context of the potential social culture based on “Rule by Man,” it is of great significance to study the emotional orientation variables of employees. Therefore, the affective commitment dimension of organizational commitment is selected to explore and analyze the relationship between ambidextrous leadership and workplace deviance behavior. Morris and Sherman [55] suggest that leadership behaviors such as encouraging cooperation, providing feedback, and recognizing contributions of employees are conducive to improve their organizational commitment. Some organizational factors (e.g., leader’s trust, superior support, etc.) have a positive effect on affective commitment of employee. According to the above, we posit that ambidextrous leadership is an important indicator to predict employee’s affective commitment.

The influence of affective commitment in an organization is mainly reflected in organizational effectiveness, employee performance, and citizenship behavior, which stem from the change of affective commitment to employee behavior in terms of intrinsic values and psychological attitudes [56]. Wollard and Shuck [57] suggest that affective commitment has a positive correlation with employee engagement. That is, employees with high affective commitment have stronger job engagement, while employees with low affective commitment will have a significant reduction in their engagement. The extant literature depicts organizational commitment as an antecedent variable for employee citizenship behavior, and affective commitment plays a predictive role in organizational citizenship behavior [58]. However, not all dimensions of organizational commitment have benefits to organizational effectiveness. Prior studies have proved the positive relationship between affective commitment and organizational performance [59]. Specifically, affective commitment has a strong effect on organizational effectiveness, including the negative effect on turnover rate, absenteeism rate, and negative organizational behavior [60]. Vandenberghe et al. [53] suggest that affective commitment from employees to leaders effectively reduces their turnover tendencies and negative dissatisfaction. Therefore, employee affective commitment is a significant predictive variable for organizational citizenship behavior, and it can also effectively reduce workplace deviance behavior.

Thus, according to affective events theory, the charismata and virtues of transformational leadership make employees feel the closeness and care of their organizations [26,27]. Employees will strengthen their affective commitment after feeling the comfort and trust from the organization, and then they tend to contribute to the organization rather than deviate from their work behavior. Transactional leadership emphasizes communication with employees and the achievement of agreement, which develops employees’ affective commitment through management by objectives [28]. Then, the combination of normative work and contractual spirit restricts employee workplace deviance behavior. Therefore, we hypothesize that

**Hypothesis** **2.**  *Affective commitment mediates the relationship between ambidextrous leadership and workplace deviance behavior.*

### 2.4. The Moderating Role of Supervisor–Subordinate Exchange Guanxi

Chinese employees attach great importance to personal relations in organizations. “*Guanxi*” is one of the core concepts in Chinese traditional culture and occupies an important position in Chinese social contexts [61]. *Guanxi* refers to an informal interpersonal phenomenon of private contact and is different from the formal social network [62]. Chinese *guanxi* is mainly based on the sharing of social experience, preferences, and trust between individuals [61,62]. Given that communications between leaders and immediate employees are very frequent during work, and their interpersonal distance is also close, the leadership–member relationship has been widely studied by scholars in the field of organizational behavior [40]. Chen and Tjosvold [63] highlight that supervisor–subordinate exchange *guanxi* is a bidirectional, special and emotional personal relationship. To a large extent, this relationship is cultivated, formed, maintained, and developed by informal and secret social communication activities (e. g., private meals through informal channels, greeting and visits during festivals, gift giving, caring to family members, etc.) based on the common and mutual interests of supervisors and subordinates outside the workplace [62,64].

According to social exchange theory, transformational leadership promotes employees to obtain more work resources in high-quality supervisor-subordinate exchange *guanxi*, which increases employees’ willingness to integrate themselves into their job roles [65]. They may improve their work engagement and achieve organizational performance better, thereby reducing workplace deviance behavior significantly due to good interpersonal ambiance [66]. Conversely, transformational leadership may have limited influence on employee behaviors under low quality supervisor–subordinate exchange *guanxi* and can even have the opposite effect. Transactional leadership that emphasizes the compliance with division of labor is more likely to affect employees in high-quality supervisor–subordinate exchange *guanxi*. Employees with organizational citizenship behaviors can achieve more rewards and resources, and they avoid violations and mistakes [50]. Employees are motivated to work hard under the transactional leadership through reducing workplace deviance behavior and avoiding losing rewards or being punished [67]. On the contrary, the impact of transactional leadership on employees will be restricted in low-quality supervisor–subordinate exchange *guanxi*. Group psychological deviation and the bandwagon effect may cause employees to reduce their expectations for the exchange relationship and then induce workplace deviance behavior [68]. Taken together, the impact of ambidextrous leadership (composed of transformational leadership and transactional leadership) on employee workplace deviance behavior is different in high and low-quality supervisor–subordinate exchange *guanxi*. Therefore, we propose that

**Hypothesis** **3.**  *Supervisor–subordinate exchange *guanxi* moderates the relationship between ambidextrous leadership and workplace deviance behavior.*

As an important phenomenon in Chinese society, supervisor–subordinate exchange *guanxi* is based on the fact that both sides pay close attention to reputation, affection, and organizational harmony [62,63,64]. *Guanxi* promotes the affective commitment, altruism, and responsibility of employees to engage in organizational citizenship behavior [62,63]. The quality of supervisor–subordinate exchange *guanxi* is measured by trust and loyalty, which can facilitate the development of ownership and organizational citizenship behavior of employees and promote communication and interaction between supervisors and subordinates through formal and informal occasions [69]. Frequent communication deepens employees’ understanding to leadership preferences and value orientations. Moreover, the role models of transformational leadership are observed and imitated by employees and may promote shared values and goals between employees and leaders [70]. The optimism and perseverance of employees can enhance their confidence and ability to finish their tasks and even form a strong motivation to further improve their job. Transactional leaders and employees attach importance to their affection component in high-quality supervisor–subordinate exchange *guanxi*, so their exchange result is fair and satisfactory [71]. The fairness and satisfaction will further promote affection between leaders and employees, produce respect and trust, and thus stimulate more affective commitment of employees. Therefore, we hypothesize that

**Hypothesis** **4.**  *Supervisor–subordinate exchange *guanxi* moderates the relationship between ambidextrous leadership and affective commitment.*

In summary, according to the suggestion of Preacher and Rucker [72], we propose a moderated mediating model that examines the indirect effect of ambidextrous leadership on employees’ workplace deviance behavior via affective commitment is affected by supervisor–subordinate exchange *guanxi*. Then, we hypothesize that

**Hypothesis** **5.**  *Supervisor–subordinate exchange *guanxi* moderates the indirect effect between ambidextrous leadership and workplace deviance behavior through affective commitment.*

**Figure 1:**
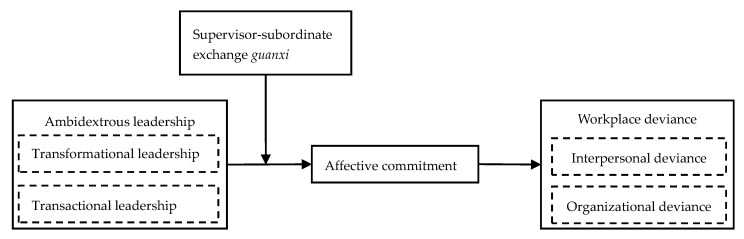
The conceptual model of relationship between ambidextrous leadership, affective commitment, supervisor–subordinate exchange *guanxi*, and workplace deviance behavior.

The proposed conceptual model (Figure 1) as follows:

## 3. Method

### 3.1. Participants and Procedures

The questionnaire was distributed among high-tech firms (including new energy, electronic communications, automobile manufacturing, and software development). The technological changes of these organizations develop faster than other industries, and the competition is more intense. These firms are in Nanjing and Shanghai. The research process proceeded as follows: First, the research team arrived at each company and communicated with the human resource (HR) director, who agreed to support our research. Second, with the assistance of the HR director and the departmental managers (such as marketing, sales, production, etc.), 10–20 employees were randomly selected from the employee list to receive the questionnaires. Finally, the employees were invited to complete the questionnaire separately and independently. The front page of the questionnaire explained the research purpose and the nature of participation as voluntary and emphasized anonymity and confidentiality during the data collection and post–data collection.

In order to control common method biases [73], the data collection involved three stages, with a two-month interval between every two consecutive measurements. Specifically, the first stage (Time 1) of data collection was realized in June 2019, where 280 questionnaires were filled out by employees, including personal information, firm description and ambidextrous leadership scale. We obtained 247 effective questionnaires after we have eliminated the incomplete, abnormal and invalid questionnaires, and the valid response rate was 88.21%. The second stage (Time 2) of data collection was in September 2019, when the employees who had completed the effective questionnaire at Time 1 filled out the affective commitment scale and supervisor–subordinate exchange *guanxi* scale. Then, 223 valid questionnaires were received, and the valid response rate was 91.28%. The third stage (Time 3) was in December 2019. The employees that have provided completed valid data from the second stage continued the survey. Then, 216 valid questionnaires were received (a response rate of 96.86%). Because the research process was spread over a long time and some employees left or refused to continue the survey, we finally collected a total of 216 complete and effective questionnaires, and the overall response rate was 77.14%.

### 3.2. Measures

The study used measuring instruments from prior studies and the responses for all items ranged from 1 (“strongly disagree”) to 5 (“strongly agree”) on a Likert scale.

The measurement of ambidextrous leadership referenced the binary operation method and was calculated by the “product formula.” According to the research on leadership contradictory behavior strategy of Gebert et al. [74], we measured ambidextrous leadership by calculating the average score of all items of transformational leadership and transactional leadership. A number of scholars have used this method to measure ambidextrous leadership and reported good reliability [75,76]. The transformational leadership scale was adapted by Li and Shi for the Chinese context [77]. We performed exploratory and confirmatory factor analyses on two separate data sets that used the original 26 items measure. The analyses revealed twenty-three items was tally with our interest requirements and the sample items included “excellent leadership skills”. The Cronbach’s alpha calculated for the scale was 0.91. Transactional leadership scale was adapted from meta-analysis of Judge et al. [27] and study of Lee et al. [78]. They removed the dimension of passive exemplary management and used contingency rewards (four items) and active exemplary management (four items) to measure transactional leadership. The scale consisted of eight items and the sample items included “My leader offered me assistance to obtain my goals.” The Cronbach’s alpha calculated for the scale was 0.88.

Meyer and Allen [79] developed the organizational commitment scale, and affective commitment is one of its three dimensions. The affective commitment scale consisted of six items and the sample items included “I am willing to keep on working in the present organization.” The Cronbach’s alpha was 0.89.

The supervisor–subordinate exchange *guanxi* instrument was adapted from Law et al. [80] and consisted of six items. The sample items included “I know and care about the family and job of my boss.” The Cronbach’s alpha was 0.78.

Workplace deviance has interpersonal deviance and organizational deviance [12]. Interpersonal deviance scale was adapted from Bennett and Robinson [81] and consisted of six items. The sample items included “playing pranks on colleagues at the workplace.” The Cronbach’s alpha calculated for the scale was 0.87. Organizational deviance scale was adapted from Bennett and Robinson [81] and also consisted of six items. The sample items included “intentionally working slower on assigned tasks.” The Cronbach’s alpha calculated for the scale was 0.92.

Based on previous studies of organizational behavior field, we controlled for age, gender, education, and work seniority [82]. Gender was coded as 0 = female and 1 = male, and education was coded 1 = high school or below, 2 = bachelor degree, 3 = Master’s degree, 4 = Master’s degree or above.

### 3.3. Validity Analyses

As the survey is a self-evaluation for employees, it is necessary to test the common method biases and multicollinearity of the data. First, we used the Harman monofactor analysis test to analyze the common method biases of the sample data, and the unrotated monofactor interpretation variable was 19.33%, which did not account for half of the total variance explained. Second, after centralizing the data of each variable, the tolerance range is 0.45 to 0.87, and the variance inflation factor is less than 3.0. Therefore, it can be determined that there is not multicollinearity problem between the variables. Furthermore, the study did not have the problem of any retrospective biases, as the items included in the questionnaire focused on the activities, events, and outcomes in relation to the perceptions of the respondents, which helped reduce the potential, if any, for retrospective bias in this study.

To observe factor structure of the variables, we conducted confirmatory factor analysis (CFA) using AMOS 24.0 (SPSS Inc., Chicago, IL, USA) with maximum likelihood estimation procedures. The discriminant validity of each scale was tested by comparing χ^2^/df, CFI, TLI, IFI, RMSEA, and other comparison indices. As shown in Table 1, compared with other solutions, the expected six-factor solution (transformational leadership, transactional leadership, affective commitment, supervisor–subordinate exchange *guanxi*, interpersonal deviance, and organizational deviance) displayed adequate fit with the data (χ^2^/df = 2.02, *p* < 0.01; TLI = 0.92; CFI = 0.93; IFI = 0.93; RMSEA = 0.07, *p* < 0.01). Therefore, the results suggest that these factors—transformational leadership, transactional leadership, affective commitment, supervisor-subordinate exchange *guanxi*, interpersonal deviance, and organizational deviance—measured unique constructs. Furthermore, the CFA results of transformational leadership structure and transactional leadership structure are displayed separately in Table 1.

## 4. Results

According to the results of descriptive statistics, the 216 participants consisted of 123 males and 93 females whose combined average age was 32.58 years. Male employees are slightly more common than female employees, so the proportion of men to women is reasonable. There is little difference between the number of employees aged 35 and under (about 54.2%) and those aged 36 and over (about 45.8%). Young people account for a small majority, indicating that the middle-aged and young employees are the main roles of production and management activities. Regarding educational background, most of the subjects have a bachelor degree (about 38.9%) or a Master’s degree (about 48.6%). It may be related to the districts and industry attributes of the sample firms, because the study took high-tech enterprises as its main research objects. Furthermore, most employees have less than 10 years of tenure (about 85.9%), and more than half of them (about 61.9%) have worked for less than five years. The above descriptive data show that young and middle-aged employees are the main workforces in their organizations, and it is necessary and urgent to develop research on the working environment, affective psychology, the relationship between organizations and leaderships, and workplace behavior in order to retain them.

Table 2 presents descriptive statistics and correlations for all the variables in the study. As predicted, ambidextrous leadership is negatively related to interpersonal deviance (r = −0.29, *p* < 0.01) and organizational deviance (r = −0.27, *p* < 0.01), and it is positively related to affective commitment (r = 0.26, *p* < 0.01). Moreover, supervisor–subordinate exchange *guanxi* did not relate significantly to ambidextrous leadership, interpersonal deviance and organizational deviance. Therefore, hypothesis 1 is initially supported.

Table 3 provides results, analyzed by SPSS software (SPSS Inc., Chicago, IL, USA) and the PROCESS program (SPSS Inc., Chicago, IL, USA) developed by Hayes [83], for mediated impact and moderated impact of the model. First, we verified the mediating effect with regression analysis and model 4 of the PROCESS program. Following the step of Baron and Kenny [84] for hierarchical regression, the result revealed that ambidextrous leadership is negatively related to organizational deviance (M1, β = −0.04, *p* < 0.01) and interpersonal deviance (M3, β = −0.19, *p* < 0.01). After adding affective commitment into the model as mediator, ambidextrous leadership still had a negative effect on organizational deviance (M2, β = −0.02, *p* < 0.05) and interpersonal deviance (M4, β = −0.12, *p* < 0.05), and the affective commitment also had a negative significant effect on organizational deviance (M2, β = −0.24, *p* < 0.01) and interpersonal deviance (M4, β = −0.22, *p* < 0.01). The regression coefficient changes little, and it concludes that affective commitment has a partial mediating effect on the relationship between ambidextrous leadership and organizational deviance as well as ambidextrous leadership and interpersonal deviance. Hypothesis 2 was partially accepted. Furthermore, the results of the Sobel test indicated that affective commitment indirectly and significantly mediates the relationship of ambidextrous leadership with organizational deviance (effect = −0.02, SE = 0.04, z = −3.13, *p* < 0.01) and interpersonal deviance (effect = −0.13, SE = 0.05, z = −3.09, *p* < 0.01). 99% confidence intervals that exclude zero also provide evidence of significant indirect effects (organizational deviance: [−0.44,−0.06]; interpersonal deviance: [−0.42,−0.07]). Therefore, Hypothesis 2 was supported.

Second, we verify the moderating effect with regression analysis and model 1 by making use of the PROCESS program. In the middle of Table 3, the product term of ambidextrous leadership and supervisor–subordinate exchange *guanxi* is significantly related to organizational deviance behavior (M5, β = −0.06, *p* < 0.01, △R^2^ = 0.25) and interpersonal deviance (M6, β = −0.05, *p* < 0.01, △R^2^ = 0.32). Hypothesis 3 was supported with a significant interaction term on the ambidextrous leadership to workplace deviance behavior path. The product term of ambidextrous leadership and supervisor–subordinate exchange *guanxi* is also significantly related to affective commitment (M7, β = 0.07, *p* < 0.01, △R^2^ = 0.54). Hypothesis 4 was supported, too.

**Figure 2:**
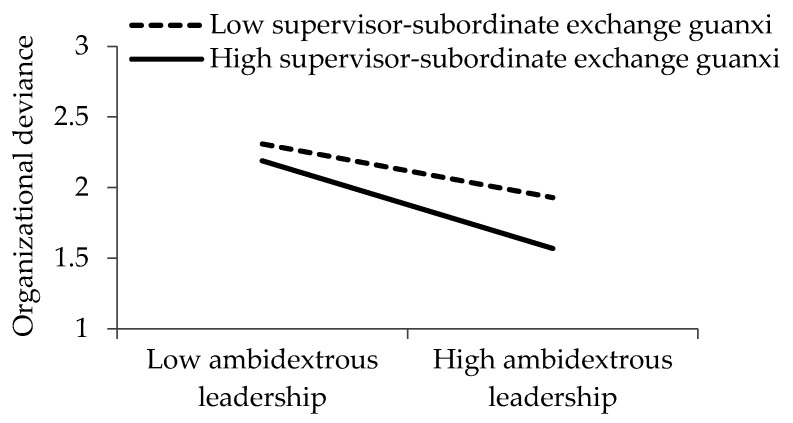
Influence of ambidextrous leadership on organizational deviance under different supervisor–subordinate exchange *guanxi* scenarios.

**Figure 3:**
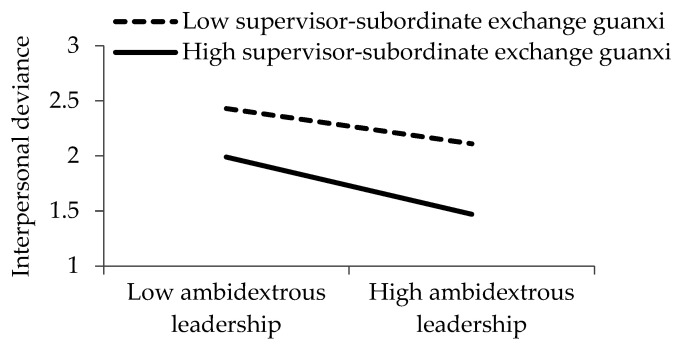
Influence of ambidextrous leadership on interpersonal deviance under different supervisor–subordinate exchange *guanxi* scenarios.

**Figure 4:**
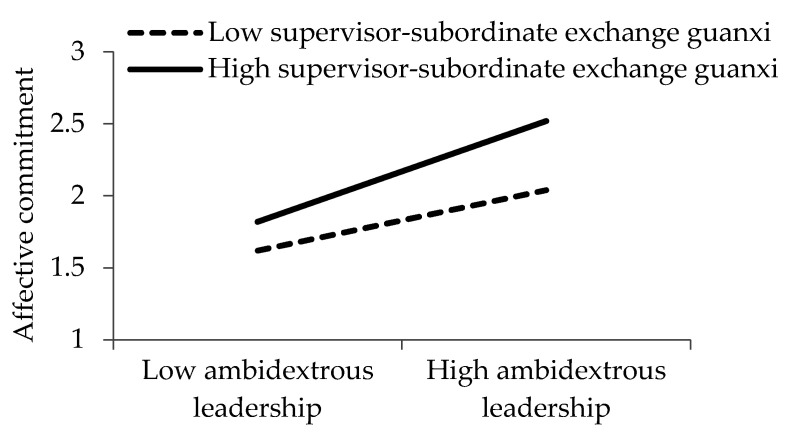
Influence of ambidextrous leadership on affective commitment under different supervisor–subordinate exchange *guanxi* scenarios.

Figure 2, Figure 3 and Figure 4 demonstrate the interactive effects of ambidextrous leadership and supervisor–subordinate exchange *guanxi*. Based on the procedure recommended by Cohen et al. [85], we visualized the influence of ambidextrous leadership on organizational deviance, interpersonal deviance, and affective commitment with the following two different supervisor–subordinate exchange *guanxi* scenarios: one standard deviation higher and one standard deviation lower than the average.

Finally, we applied model 8 of the SPSS PROCESS macro to analyze the indirect effect of ambidextrous leadership on organizational deviance and interpersonal deviance through affective commitment under different degrees of supervisor–subordinate exchange *guanxi* [69]. At the bottom of Table 3, the index of moderated mediation indicates that any two conditional indirect effects defined by different supervisor–subordinate exchange *guanxi* scenarios are different statistically. For organizational deviance (index = −0.03, SE = 0.01, 95%CI = [−0.07, −0.01]), the mediating role of affective commitment between ambidextrous leadership and organizational deviance is not significant for low (effect = −0.38, SE = 0.01, 95%CI = [−0.02, 0.06], contain zero) and is significant for high (effect = −0.05, SE = 0.02, 95%CI = [−0.13, −0.03], without zero) levels of supervisor-subordinate exchange *guanxi*. For interpersonal deviance (index = −0.06, SE = 0.02, 95%CI = [−0.06, −0.01]), the mediating role of affective commitment between ambidextrous leadership and interpersonal deviance is not significant for low (effect = −0.18, SE = 0.04, 95%CI = [−0.13, 0.04], contain zero) and is significant for high (effect = −0.23, SE = 0.05, 95% CI = [−0.30, −0.22], without zero) levels of supervisor–subordinate exchange *guanxi.* These results indicate that the indirect effect between ambidextrous leadership and workplace deviance behavior (both organizational deviance and interpersonal deviance) is conditional upon supervisor–subordinate exchange *guanxi*, such that higher levels of exchange *guanxi* increase the magnitude of the indirect effect. Therefore, the proposed moderated mediation Hypothesis 5 is supported.

## 5. Discussion

### 5.1. Theoretical Implicationss

Based on the perspective of affective and “*guanxi*,” the study shows clearly the action process of transformational leadership and transactional leadership on employees’ subsequent negative workplace behavior and its contingency influence mechanism.

First, although scholars believe that “affective is one of the essential factors that drive people’s behavior,” the potential mediating mechanism developed by affective in the relationship between leadership and employees’ negative organizational behavior has not been paid enough attention [6,16]. Our research constructs an integrated multi-theory-driven model to examine how ambidextrous leadership affects individual psychological motivation and behavioral responses. The influence of transformational leadership and transactional leadership on workplace deviance behaviors is discussed from the perspective of emotional commitment. In other words, the ambidextrous leadership of transformational leadership and transactional leadership promotes employees’ affective commitment to reduce their workplace deviance behaviors. We extend the domain of leadership style that affects employees’ negative organizational behavior and motivates managers to rethink their relationship with subordinates in order to avoid employee workplace deviance behavior. Meanwhile, our study also responds to the call for more research on the impacting factors of employee deviance behavior in the organization [86,87].

Second, the study takes supervisor–subordinate exchange *guanxi* as the situational factor, which breaks the inherent cognition of the boundary condition of the relationship between the leadership style and the employees’ negative organizational behavior in previous studies and also promotes the emergence of new research ideas in this field. Turnley and Feldman [88] suggest that the perception of employees to organization comes largely from their supervisor because the affective attachment of employees to the organization is usually conveyed by their direct leader. That is to say, the relationship between supervisor and subordinate in ambidextrous leadership will have an impact on employees’ affective commitment and their workplace behavior. It is also in accordance with the feature of supervisor–subordinate exchange *guanxi* in Chinese context. Our research confirmed that the supervisor–subordinate exchange *guanxi* and ambidextrous leadership had a significant interaction effect on employee workplace deviance behavior and affective response. The results contribute new knowledge to the recently emerging research field on linking ambidextrous leadership and employees’ negative organizational behavior. The findings also reveal that the interaction effect of supervisor–subordinate exchange *guanxi* and ambidextrous leadership on employees’ workplace deviance behavior and complement new idea and empirical experience in related fields.

### 5.2. Practical Implications

The study indicates how to human resources practice through restraining and reducing the workplace deviance behavior of employees. The following implications are drawn from this study:

First of all, leaders can use agile management to promote the development and effectiveness of leadership style. By integrating the strengths of transformational leadership and transactional leadership, the combined effect of leadership is far more powerful than single leadership. Specifically, ambidextrous leadership may improve the managerial skills of leaders and deepen the affective entropy of employees through the development of an employee-oriented management system to help employees meet job demands. Meanwhile, based on the accurate identification of employees’ demands, managers are able to show solicitude for employees and employ motivational techniques to engage employees and get them to work have, which should result in the increase of employees’ positive organizational behavior and effectively reduce their workplace deviance behavior.

Second, leaders need to focus on the affective state and behavioral motivation of employees for anticipating and preventing the occurrence of workplace deviance behavior. The leadership style should exemplify a greater sense of fairness and openness and pay attention to building and maintaining employees’ perceived organizational support. Moreover, positive emotion (such as affective commitment) of employees should be cultivated and enhanced, through flexible leadership strategies, and then their job satisfaction may be improved, and workplace deviance behavior may be restrained.

Finally, organizational leaders should concentrate on the influence of situational factors and establish harmonious and healthy internal relationships in the organization. The organization must recognize the contribution of staff to their company in order to strengthen the leadership effectiveness of supervisors and the interpersonal skills of subordinates. Leaders and employees can promote their personal communication and interaction for building a supportive and trustful relationship structure. Simultaneously, any leadership training project should be carried out actively to enhance the understanding of harmful results of workplace deviance behavior, and leaders need more practical ability to develop positive, continuous and consistent actions for supervising subordinates by means of *guanxi*.

### 5.3. Limitations and Future Research Directions

Although the current study has made important contributions to the research on supervisor–employee relations, we should mind the limitations. Future studies could include the following aspects. First, the samples were taken from high-tech industries in Nanjing and Shangha, because these companies are representative to verify the model of our study. However, the data’s lack of comparative research for different regions, different industries, and different types of firms, which limits the external validity of our results. Future research could collect data from multiple cities (or provinces/geographical regions), diversified industries, and manifold type of firms and then form targeted and detailed guidance for practitioners. Second, the study focuses on the impacts of employees’ direct supervisors on their workplace deviance behavior. However, leaders at 2–5 different levels, from employees’ direct supervisors to executives, will influence employees’ perception and behavior directly or indirectly [89]. In the modern workflow and hierarchical organization structure, how and why multi-level leaders affect employee behavior depends on their abilities to deal with strategic exceptional events and solve uncertainties in a specific working condition [90]. Therefore, we suggest that future studies could further explore the influence and difference of multi-level and multivariate leadership style on employees’ workplace deviance behavior.

## 6. Conclusions

Drawing on affective events theory and social exchange theory, we posit that a high-quality supervisor–subordinate relationship affects the levels of productivity and performance in the workplace. Furthermore, this interactive and mutual relationship has a significant impact on the task performance of employees who display workplace deviance behavior, and our study supported the above. In the first place, there is a significant negative correlation between ambidextrous leadership and workplace deviance behavior (both organizational deviance and interpersonal deviance). The result is in line with previous studies that found that ambidextrous leadership can stimulate the positive behavior of employees and reduce their negative organizational behavior [22,23]. In the second place, our study suggests that affective commitment mediates the influence of ambidextrous leadership on workplace deviance behavior. This mediation process means that transformational leadership and transactional leadership are related to higher affective commitment, and higher affective commitment is related to lower workplace deviance behavior. Meanwhile, this mediate effect is regulated by the supervisor–subordinate exchange *guanxi*. Specifically, with high-quality supervisor–subordinate exchange *guanxi*, ambidextrous leadership is more likely to enhance employee affective commitment and then improve their performance and satisfaction. The motivation for employees to engage in workplace deviance behaviors is also minimized. Meanwhile, with low-quality supervisor–subordinate exchange *guanxi*, ambidextrous leadership has difficulty in gaining employees’ trust, and the impact of affective commitment to discourage employee workplace deviance behavior is greatly reduced.

## Figures and Tables

**Table 1:** The results of confirmatory factor analysis.

Measurement Models		χ^2^(df)	Δχ^2^(Δdf)	CFI	TLI	IFI	RMSEA
six-factor	Tf,Ta,Ac,Gx,Id,Od	277.21(137) **	-	0.93	0.92	0.93	0.07
five-factor	Tf + Ta, Ac,Gx, Id,Od	404.01(142) **	126.80(5) **	0.87	0.85	0.88	0.09
five-factor	Tf,Ta, Ac,Gx, Id + Od	595.54(142) **	318.33(5) **	0.88	0.84	0.88	0.10
four-factor	Tf + Ta,Ac,Gx,Id + Od	722.83(146) **	445.62(9) **	0.82	0.80	0.82	0.11
three-factor	Tf + Ta,Ac + Gx,Id + Od	993.90(149) **	716.69(12) **	0.79	0.73	0.70	0.16
two-factor	Tf + Ta + Ac,Gx + Id + Od	1309.23(151) **	1032.02(14) **	0.54	0.56	0.54	0.19
**Transformational Leadership**							
four-factor	Tf1,Tf2,Tf3,Tf4	576.20(233) **	-	0.91	0.90	0.91	0.06
three-factor	Tf1 + Tf2,Tf3,Tf4	1265.69(237) **	689.49(4) **	0.89	0.86	0.87	0.10
two-factor	Tf1 + Tf2,Tf3 + Tf4	1749.25(241) **	1173.05(8) **	0.75	0.74	0.76	0.13
one-factor	Tf1 + Tf2 + Tf3 + Tf4	2349.19(245) **	1772.99(12) **	0.68	0.64	0.66	0.19
**Transactional Leadership**							
two-factor	Ta1,Ta2	206.89(41) **	-	0.96	0.95	0.96	0.06
one-factor	Ta1 + Ta2	334.45(43) **	127.56(2) **	0.88	0.81	0.85	0.08

**Note:** Tf = transformational leadership, Ta = transactional leadership, Ac = affective commitment, Gx = supervisor–subordinate exchange *guanxi*, Id = interpersonal deviance, Od = organizational deviance. “+” means two factors are combined into one factor. ** *p* < 0.01.

**Table 2:** Means, standard deviation, and correlations of variables.

		M	SD	1	2	3	4	5	6	7	8	9
1	Gender	1.57	0.49	—								
2	Age	32.40	0.83	−0.11	—							
3	Education	2.56	0.71	0.07	0.03	—						
4	Working seniority	4.58	0.91	−0.12	0.31 **	−0.19 **	—					
5	ambidextrous leadership	13.45	4.59	0.08	0.05	0.00	0.11	—				
6	affective commitment	3.77	0.82	0.02	0.17	0.10	0.21	0.26 **	—			
7	supervisor-subordinate exchange guanxi	3.47	0.68	0.10	0.17 *	0.01	0.14 *	0.01	0.14 *	—		
8	organizational deviance	2.51	0.76	0.16 *	−0.11	−0.04	−0.15	−0.27 **	−0.25 **	−0.08	—	
9	interpersonal deviance	2.16	0.74	0.17 *	−0.13	−0.00	−0.21	−0.29 **	−0.23 **	−0.12	0.21 **	—

**Note:** M = mean; SD = standard deviation. ** *p* < 0.01;* *p* < 0.05.

**Table 3:** The process analysis results of hierarchical regression and conditional effect test.

The Mediating Effect Test								
The Mediating Results		*β*	*SE*	*t*	*p*	*R^2^*	*△R^2^*	*F*
M1	Ambidextrous leadership → Organizational deviance	−0.04 **	0.06	3.95	0.00	0.11	0.07	5.12 **
M2	Ambidextrous leadership → Organizational deviance Affective commitment → Organizational deviance	−0.02 * −0.24 **	0.04 0.05	4.18 8.73	0.03 0.00	0.35	0.24	18.50 **
M3	Ambidextrous leadership → Interpersonal deviance	−0.19 **	0.07	4.15	0.00	0.12	0.09	5.45 **
M4	Ambidextrous leadership → Interpersonal deviance Affective commitment → Interpersonal deviance	−0.12 * −0.22 **	0.05 0.05	6.23 8.35	0.01 0.00	0.33	0.22	17.64 **
**Sobel Test Results**	**Effect**	**SE**	**LL 95% CI**		**UL 95% CI**		***z***	***p***
Ambidextrous leadership → Organizational deviance Ambidextrous leadership → Interpersonal deviance	−0.02 −0.13	0.04 0.05	−0.39 −0.31		−0.11 −0.13		−3.13 −3.09	0.00 0.00
Conditional Indirect Effect Results	Effect	SE	LL 99% CI		UL 99% CI			
Ambidextrous leadership → Organizational deviance Ambidextrous leadership → Interpersonal deviance	−0.02 −0.12	0.05 0.06	−0.44 −0.42		−0.06 −0.07			
**The Moderating Effect Test**								
**The Moderating Results**		***β***	**SE**	***t***	***p***	***R^2^***	***ΔR^2^***	***F***
M5	Ambidextrous leadership → Organizational deviance S-s exchange *guanxi* → Organizational deviance Ambidextrous leadership × S-s exchange *guanxi* → Organizational deviance	−0.25 ** −0.12 ** −0.06 **	0.06 0.11 0.04	4.22 3.07 3.53	0.00 0.00 0.00	0.16	0.25	5.76 **
M6	Ambidextrous leadership → Interpersonal deviance S-s exchange *guanxi* → Interpersonal deviance Ambidextrous leadership × S-s exchange *guanxi* → Interpersonal deviance	−0.21 ** −0.27 * −0.05 **	0.06 0.10 0.03	5.33 3.98 8.29	0.00 0.02 0.00	0.15	0.32	5.25 **
M7	Ambidextrous leadership → Affective commitment S-s exchange *guanxi* → Affective commitment Ambidextrous leadership × S-s exchange *guanxi* → Affective commitment	0.28 ** 0.17 ** 0.07 **	0.07 0.25 0.02	4.40 6.28 3.70	0.00 0.00 0.00	0.18	0.54	6.56 **
**The Moderated Mediation** **Effect Test**								
**The Bootstrapping Results of Conditional Indirect Effect**		**Effect**		**SE**	**LL 99% CI**		**UL 99% CI**	
Ambidextrous leadership → Organizational deviance	Low (–1 SD) High (+1 SD)	−0.38 −0.05		0.01 0.02	−0.02 −0.13		0.06 −0.03	
The results of moderating mediation	Low (–1 SD) High (+1 SD)	−0.03		0.01	−0.07		−0.01	
Ambidextrous leadership → Interpersonal deviance		−0.18 −0.23		0.04 0.05	−0.13 −0.30		0.04 −0.22	
The results of moderating mediation		−0.06		0.02	−0.06		−0.01	

**Note:** ** *p* < 0.01; * *p* < 0.05; bias-corrected CI is reported; bootstrapping sample size = 5000; low = 1 SD below the mean; high = 1 SD above the mean.
